# Working hours are closely associated with depressive mood and suicidal ideation in Korean adults: a nationwide cross-sectional study

**DOI:** 10.1038/s41598-021-02574-8

**Published:** 2021-11-29

**Authors:** Sangsoo Han, Yujin Ko, Ji Eun Moon, Young Soon Cho

**Affiliations:** 1 Department of Emergency Medicine, Soonchunhyang University Bucheon Hospital, 170 Jomaru-ro, Bucheon, 14584 Republic of Korea; 2 Department of Psychiatry, Soonchunhyang University Bucheon Hospital, 170 Jomaru-ro, Bucheon, 14584 Republic of Korea; 3 Department of Biostatistics, Clinical Trial Center, Soonchunhyang University Bucheon Hospital, 170 Jomaru-ro, Bucheon, 14584 Republic of Korea

**Keywords:** Health care, Health occupations, Risk factors

## Abstract

Long working hours have been presumed to negatively influence health. However, evidence is lacking regarding any associations of working hours with depressive mood or suicidal ideation. We investigated the relationships of working hours with depressive mood and suicidal ideation in a representative sample of the Korean general population. We analyzed data collected by the Korea National Health and Nutrition Examination Surveys VI and VII (2013–2018). Depressive mood and suicidal ideation were identified through self-reporting. We divided participants into four groups according to weekly working hours: 30–40, 41–50, 51–60, and > 60 h/week. Sampling weights were applied to obtain estimates for the general Korean population. We analyzed 14,625 participants, of whom 5383 (36.8%), 4656 (31.8%), 2553 (17.5%), and 2033 (13.9%) worked 30–40, 41–50, 51–60, and > 60 h/week, respectively. In these groups, 3.6%, 4.4%, 5.2%, and 6.3% of the participants reported depressive mood, while 1.8%, 1.9%, 2.2%, and 3.6% reported suicidal ideation. In multiple regression analyses, compared with the 30–40 h/week group, the adjusted odds ratios of the 41–50, 51–60, and > 60 h/week groups for depressive mood were 1.35 (1.08–1.69), 1.5 (1.14–1.97), and 1.6 (1.19–2.14). A similar trend was evident for suicidal ideation (odds ratios 1.16 [0.82–1.63], 1.48 [0.99–2.21], and 2.29 [1.53–3.42]). Long working hours are significantly associated with depressive mood and suicidal ideation.

## Introduction

Long working hours are associated with negative health effects; many countries worldwide seek to limit working hours. In most European countries, the legal limit is fewer than 48 h per week; approximately half of all European countries impose a 40-h limit^1^. However, approximately one-third of the world's workforce still works more than 48 h per week^2^. Among Organization for Economic Co-operation and Development (OECD) member countries, Korea has the third longest working week worldwide (after Mexico and Costa Rica)^3^. In 2019, Korean working hours were 1967 annually, thus 241 h more than the mean of 1726 h among the 35 OECD countries.

Long working hours are closely associated with hypertension, diabetes, cardiovascular disease, and stroke^4–6^. Long working hours also affect mental health, such that they cause increasing fatigue and distress^7^. Such negative effects are manifested in several manners, including depressive mood and suicidal behavior that can cause injury and hospitalization, thereby imposing societal burdens of billions of dollars^8,9^.

Suicidal ideation is one of the strongest predictors of suicidal death and suicide attempts can have negative consequences such as injury and hospitalization, even if it does not end in death^9^. Various efforts have been made to explore the psychological processes in the development of suicide ideation and suicidal behavior^10–12^. The integrated motivational-volitional (IMV) model of suicidal behavior provides an explanation of the development of suicidal ideation. Suicidal ideation and intent are described as occurring when a person feels that he or she is subjectively trapped in a hopeless situation (entrapment)^11^. Entrapment consists of a two-dimensional construct: an external entrapment describes external conditions (e.g., obligations, work problems) from which one feels trapped and wants to escape, and an internal trap describes one's own internal limitations and painful thoughts that one feels unable to escape^13^.

Because workplace problems can act as an external entrapment that can generate suicidal ideation, there have been many studies on the association between work environment and suicidal behavior^14–16^. Several studies found that long working hours have negative effects on mental health (e.g., depressive mood and suicidal ideation), but only a few were large-scale studies using nationwide databases^8,17,18^. Mental health problems are also affected by socioeconomic and environmental factors, which must thus be considered^19,20^. Therefore, using a nationwide survey database that includes socioeconomic and environmental factors could be of great advantage. A previous study using nationwide survey data in Korea reported that long working hours were linked to suicidal thoughts^18^. However, the socioeconomic and environmental factors such as education level, perceived health status, and comorbidities related to suicide were not considered in the study^21,22^. We considered potential confounders including social demographic factors and health status when examining the relationships of working hours with depressive mood or suicidal ideation in a large sample of Korean adults.

Our primary purpose was to examine the associations of working hours with mental health problems, such as depressive mood (sadness or hopelessness) and suicidal ideation (seriously considered), in adults ≥ 19 years of age. Our secondary purpose was to determine the associations of mental health with working hours in subgroups stratified according to sex, shift work status, and occupation type (white-, pink-, or blue-collar).

## Materials and methods

### Study design and participants

This cross-sectional study used the databases of the 6th and 7th National Health and Nutrition Surveys (KNHANESs) conducted by the Korea Centers for Disease Control and Prevention (KCDC) from 2013 to 2018. The KNHANES has been performed annually since 1998 to evaluate the health and nutritional status of the Korean population. A national representative sample is obtained using a stratified, multistage cluster-sampling design. Participants vary among years and are not continuously monitored. The survey includes approximately 10,000 independent samples from 192 primary sampling units (PSUs) every year; each PSU is selected based on administrative districts and housing types, using a sampling frame of all census blocks containing the Korean resident registration addresses. The survey features a health interview, health examination, and nutritional survey. In this study, workers ≥ 19 years of age were analyzed. The exclusion criteria were (1) age under 19; (2) unemployed (not working for money, including students or housewives)^23^; (3) working for fewer than 30 h per week (such people are not regarded as full-time employees in Korea)^5,24^; or (4) non-completion of the working hours and/or job questionnaire(s).

### Working hours

Working hours were determined by asking: “What is your average number of hours worked per week?” Overtime work was included and mealtimes were excluded. The answers were divided into 10-h increments: 31–40, 41–50, 51–60, and > 60 h per week^5,24^.

### Depressive mood and suicidal ideation

Depressive mood was assessed by asking “Have you felt sadness or despair which hindered everyday life consistently for 2 weeks or more during the last year?” (yes or no). Suicidal ideation was assessed by asking “Have you ever seriously considered suicide in the last year?” (yes or no).

### Potential mediators and confounders

Demographic characteristics, socioeconomic status, medical histories, and lifestyle habits revealed in the health interview and examination were used in the analysis. The body mass index was the weight divided by the height squared. All participants were divided into non-smokers, ex-smokers, and current smokers. Alcohol consumption was divided into none, ≤ 1 drink/month, 2 drinks/month to 3 drinks/week, and ≥ 4 drinks/week. Educational level was divided into elementary school, middle school, high school, and college or university. Household income level was divided into quartiles. Marital status was divided into single, married, separated, separated by death, and divorced. Perceived health status was recorded as very good, good, average, bad, and very bad, depending on answers to the question “How do you perceive your health?”. Sleep duration was explored by asking “How many hours do you sleep each day?”. Weekly, aerobic physical activity was categorized by intensity: mid-intensity for ≥ 2 h 30 min, high-intensity for > 1 h 15 min, or a combination of mid- and high-intensity activity for a longer time than stated above (1 min of high-intensity activity was considered equivalent to 2 min of mid-intensity activity)^25^. Occupational types were classified as white-collar (office workers, professionals, or managers); pink-collar (service or sales workers); and blue-collar (manufacturing, construction, craft, and related workers)^26^. Shift work status was identified. Any major comorbidity such as hypertension, diabetes, dyslipidemia, stroke, myocardial infarction, angina, or a malignancy (e.g., lung, stomach, liver, colon, breast, or cervical cancer) was recorded.

### Statistical analysis

Analysis of variance was used to compare continuous variables; the data are presented as means with standard deviations. The chi-squared test was employed to analyze categorical variables; the data are presented as numbers with percentages. We used logistic regression to calculate odds ratios (ORs) with 95% confidence intervals (CIs). We constructed three models to explore potential mediators and confounders. Model 1 was unadjusted. Model 2 was adjusted for age and sex. Model 3 was fully adjusted for age, sex, smoking status, alcohol consumption, educational level, household income, marital status, perceived health status, occupation, sleep duration, physical activity, and comorbidities. We performed subgroup analysis according to sex (male or female); shift work status (no or yes); and occupation (white-, pink-, or blue-collar). As KNHANES data were derived from multistage complex probability sampling, population weights were applied to all data to ensure that they were representative of the entire Korean population. All statistical analyses were performed using IBM SPSS Statistics for Windows, version 26.0 (IBM Corp., Armonk, NY, USA). A two-sided *p*-value < 0.05 was considered to indicate statistical significance.

### Ethics statement

KNHANES VI and VII were approved by the KCDC Institutional Review Board (approval nos. 2013–12EXP-03–5C, 2018–01–03-P-A). Informed consent was obtained from all participants. The study adhered to the principles of the Declaration of Helsinki. All study procedures followed relevant guidelines and regulations.

## Results

**Figure 1:**
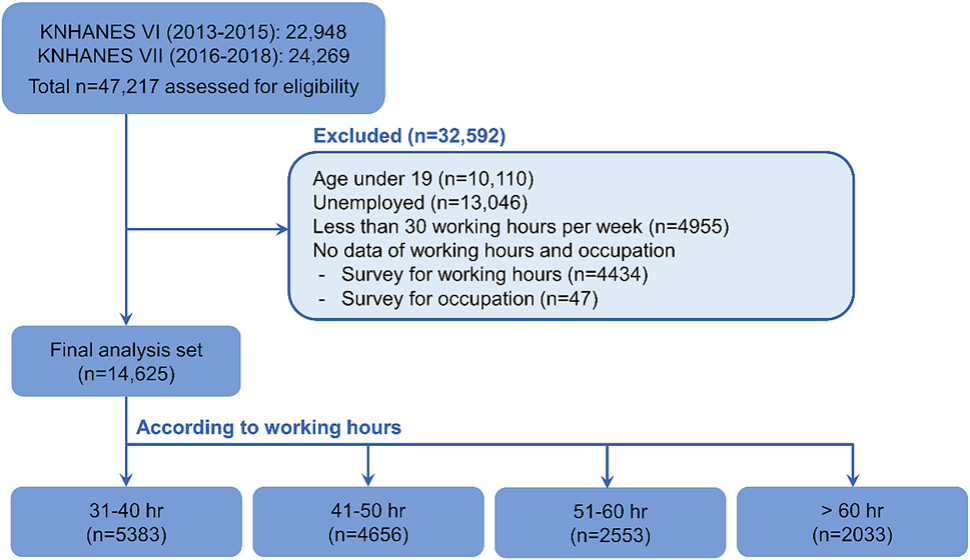
Flow chart of study participants among individuals included in the 2013 to 2018 Korea National Health and Nutrition Examination Surveys (KNHANES VI and VII).

In total, 47,217 people were surveyed. The following individuals were excluded: 9794 aged < 19 years, 13,362 who were unemployed, 4995 who were working fewer than 30 h per week, 4434 who did not report working hours, and 47 who did not report occupations. We finally analyzed 14,625 people (Fig. 1).

### General characteristics stratified according to working hours

The 14,625 participants were divided into individuals who worked 31–40 (5383; 36.8%), 41–50 (4656; 31.8%), 51–60 (2553; 17.5%), and > 60 h/week (2033; 13.9%). Participant demographic and general characteristics are shown in Table 1. Age, sex, height, weight, body mass index, smoking status, alcohol consumption, educational level, household income, marital status, perceived health status, sleep duration, aerobic physical activity, occupation, shift work, and comorbidities (except malignancy) differed significantly among the groups because the study population was very large. The depressive mood rates differed significantly among the four groups; depressive moods were reported by 196 (3.6%), 206 (4.4%), 133 (5.2%), and 128 (6.3%) participants who worked 31–40, 41–50, 51–60, and > 60 h/week, respectively (*p* < 0.001). The rates of suicidal ideation also differed significantly among the four groups: 96 (1.8%), 86 (1.9%), 55 (2.2%), and 74 (3.6%) (*p* < 0.001).

**Table 1:** General characteristics of the study population.

	31–40 h	41–50 h	51–60 h	> 60 h	*p*-value
	(n = 5383)	(n = 4656)	(n = 2553)	(n = 2033)	
Age, years	45.7 ± 13.1	44.9 ± 13.1	47.2 ± 12.8	51.3 ± 12.8	< 0.001
Sex, n (%)					< 0.001
Male	2659 (49.4)	2763 (59.3)	1688 (66.1)	1304 (64.1)	
Female	2724 (50.6)	1893 (40.7)	865 (33.9)	729 (35.9)	
Height, cm	165.1 ± 8.9	166.4 ± 9.0	166.5 ± 9.0	165.0 ± 9.4	< 0.001
Weight, kg	65.1 ± 12.4	66.8 ± 13.1	67.4 ± 12.6	67.1 ± 12.4	< 0.001
BMI, kg/m^2^	23.8 ± 3.4	24.0 ± 3.5	24.2 ± 3.4	24.6 ± 3.3	< 0.001
Smoking status, n (%)					< 0.001
Non-/ex-smoker	4252 (79.0)	3416 (73.4)	1734 (67.9)	1391 (68.4)	
Current smoker	1131 (21.0)	1240 (26.6)	819 (32.1)	642 (31.6)	
Alcohol consumption, n (%)					< 0.001
None	1004 (18.7)	752 (16.2)	450 (17.6)	463 (22.8)	
≤ 1 drink/month	1581 (29.4)	1249 (26.8)	629 (24.6)	484 (23.8)	
2 drinks/month to 3 drinks/week	2473 (45.9)	2294 (49.3)	1206 (47.2)	810 (39.8)	
≥ 4 drinks/week	325 (6.0)	361 (7.8)	268 (10.5)	276 (13.6)	
Educational level, n (%)					< 0.001
Elementary school	524 (9.7)	483 (10.4)	351 (13.8)	415 (20.4)	
Middle school	390 (7.3)	376 (8.1)	324 (12.7)	329 (16.2)	
High school	1714 (31.9)	1502 (32.3)	934 (36.6)	764 (37.6)	
College or university	2752 (51.2)	2294 (49.3)	943 (37.0)	525 (25.8)	
Household income, n (%)					< 0.001
Low	385 (7.2)	309 (6.7)	184 (7.2)	212 (10.5)	
Low-moderate	1086 (20.2)	996 (21.4)	680 (26.7)	620 (30.6)	
Moderate-high	1665 (31.0)	1569 (33.8)	862 (33.8)	616 (30.4)	
High	2238 (41.6)	1774 (38.2)	823 (32.3)	576 (28.5)	
Marital status, n (%)					< 0.001
Single	998 (18.8)	941 (20.4)	399 (15.8)	189 (9.5)	
Married	213 (4.0)	148 (3.2)	100 (4.0)	83 (4.2)	
Separated	1318 (24.8)	1018 (22.1)	521 (20.6)	424 (21.4)	
Separated by death	2306 (43.4)	2072 (45.0)	1244 (49.3)	990 (49.9)	
Divorced	483 (9.1)	424 (9.2)	260 (10.3)	299 (15.1)	
Perceived health status, n (%)					< 0.001
Very good	271 (5.0)	207 (4.5)	106 (4.2)	94 (4.6)	
Good	1605 (29.8)	1274 (27.4)	650 (25.5)	468 (23.0)	
Average	2850 (52.9)	2576 (55.3)	1451 (56.8)	1079 (53.1)	
Bad	586 (10.9)	538 (11.6)	315 (12.3)	341 (16.8)	
Very bad	71 (1.3)	61 (1.3)	31 (1.2)	51 (2.5)	
Sleep duration, h	7.1 ± 1.2	7.0 ± 1.2	6.9 ± 1.2	6.7 ± 1.3	< 0.001
Aerobic physical activity, n (%)	2019 (37.5)	1653 (35.5)	815 (31.9)	532 (26.2)	< 0.001
Occupation, n (%)					< 0.001
White-collar	2852 (32.0)	2240 (48.1)	744 (29.1)	307 (15.1)	
Pink-collar	857 (15.9)	748 (16.1)	634 (24.8)	805 (39.6)	
Blue-collar	1674 (31.1)	1668 (35.8)	1175 (46.0)	921 (45.3)	
Shift work, n (%)	612 (11.4)	596 (12.8)	392 (15.4)	453 (22.5)	< 0.001
Depressive mood, n (%)	196 (3.6)	206 (4.4)	133 (5.2)	128 (6.3)	< 0.001
Suicidal ideation, n (%)	96 (1.8)	86 (1.9)	55 (2.2)	74 (3.6)	< 0.001
Comorbidities, n (%)					
Hypertension	812 (15.1)	657 (14.1)	460 (18.0)	505 (24.8)	< 0.001
Diabetes	309 (5.7)	227 (4.9)	168 (6.6)	186 (9.2)	< 0.001
Dyslipidemia	631 (11.7)	543 (11.7)	336 (13.2)	327 (16.1)	< 0.001
Stroke	54 (1.0)	34 (0.7)	24 (0.9)	41 (2.0)	< 0.001
Myocardial infarction	24 (0.5)	30 (0.6)	16 (0.6)	25 (1.2)	0.003
Angina	46 (0.9)	41 (0.9)	26 (1.0)	42 (2.1)	< 0.001
Malignancy	65 (1.2)	36 (0.8)	25 (1.0)	26 (1.3)	0.116

Numerical parameters are expressed as means ± standard deviations and categorical parameters as counts (percentages). *BMI* body mass index.

### Relationships of working hours with depressive mood and suicidal ideation

In Model 1 (unadjusted), the ORs for depressive mood of the 41–50-, 51–60-, and > 60-h groups were higher than the OR of the 31–40-h group: 1.29 (95% CI 1.04–1.60, *p* = 0.022), 1.5 (95% CI 1.17–1.93, *p* = 0.001), and 1.8 (95% CI 1.39–2.33, *p* < 0.001), respectively. Compared with participants who worked 31–40 h, participants who worked > 60 h had a higher OR (2.39, 95% CI 1.66–3.44, *p* < 0.001) for suicidal ideation. In Model 2 (adjusted for sex and age), the ORs for depressive mood of the 41–50-, 51–60-, and > 60-h groups were higher than the OR of the 31–40-h group: 1.42 (95% CI 1.14–1.77, *p* = 0.002), 1.75 (95% CI 1.35–1.25, *p* < 0.001), and 2.03 (95% CI 1.55–2.66, *p* < 0.001). Compared with participants who worked 31–40 h, participants who worked 51–60 and > 60 h had higher ORs for suicidal ideation: 1.59 (95% CI 1.09–2.33, *p* = 0.017) and 2.7 (95% CI 1.86–3.91, *p* < 0.001), respectively. In Model 3 (adjusted for potentially confounding variables), the ORs for depressive mood of the 41–50-, 51–60-, and > 60-h groups remained higher than the OR of the 31–40-h group: 1.35 (95% CI 1.08–1.69, *p* = 0.01), 1.5 (95% CI 1.14–1.97, *p* < 0.001), and 1.6 (95% CI 1.19–2.14, *p* < 0.001). A dose–response relationship was evident between increased working hours and the risk of depressive mood. Compared with participants who worked 31–40 h, participants who worked > 60 h (OR 2.3, 95% CI 1.54–3.45, *p* < 0.001) exhibited the strongest association with suicidal ideation (Table 2).

**Table 2:** Relationships of work-related factors with depressive mood and suicidal ideation.

	Model 1			Model 2			Model 3		
	OR	95% CI	*p*-value	OR	95% CI	*p*-value	OR	95% CI	*p*-value
**Depressive mood**									
Sex									
Male	1			1			1		
Female	1.78	1.60–1.98	< 0.001	2.19	1.84–2.62	< 0.001	2.38	1.87–.3.02	< 0.001
Occupation									
White-collar	1			1			1		
Pink-collar	1.76	1.44–2.15	< 0.001	1.58	1.22–2.03	< 0.001	1.34	1.01–1.79	0.044
Blue-collar	1.71	1.42–2.07	< 0.001	1.81	1.45–2.27	< 0.001	1.41	1.08–1.85	0.013
Shift work									
No	1			1			1		
Yes	1.04	0.85–1.28	0.681	0.95	0.73–1.23	0.682	0.89	0.68–1.16	0.387
Working hours									
31–40	1			1			1		
41–50	1.29	1.04–1.60	0.022	1.42	1.14–1.77	0.002	1.34	1.06–1.68	0.013
51–60	1.50	1.17–1.93	0.001	1.75	1.35–2.25	< 0.001	1.49	1.13–1.96	0.004
> 60	1.80	1.39–2.33	< 0.001	2.03	1.55–2.66	< 0.001	1.61	1.20–2.16	0.002
**Suicidal ideation**									
Sex									
Male	1			1			1		
Female	1.67	1.43–1.94	< 0.001	2.36	1.76–3.15	< 0.001	2.80	1.97–3.97	< 0.001
Occupation									
White-collar	1			1			1		
Pink-collar	1.46	1.07–1.99	0.017	1.48	0.99–2.18	0.050	0.53	0.33–0.87	0.011
Blue-collar	1.97	1.51–2.58	< 0.001	2.61	1.77–3.86	< 0.001	0.99	0.65–1.52	0.986
Shift work									
No	1			1			1		
Yes	1.13	0.86–1.49	0.370	1.13	0.79–1.62	0.489	1.04	0.70–1.53	0.86
Working hours									
31–40	1			1			1		
41–50	1.13	0.81–1.59	0.471	1.24	0.89–1.73	0.210	1.15	0.82–1.63	0.418
51–60	1.38	0.94–2.01	0.101	1.59	1.09–2.33	0.017	1.48	0.99–2.21	0.056
> 60	2.39	1.66–3.44	< 0.001	2.70	1.86–3.91	< 0.001	2.30	1.54–3.45	< 0.001

*CI* confidence interval, *OR* odds ratio. Model 1: Unadjusted. Model 2: Adjusted by age and sex. Model 3: Fully adjusted by age, sex and environmental factors: Smoking status, alcohol consumption, educational level, household income, marital status, perceived health status, occupation, sleep duration, physical activity, and comorbidities.

### Subgroup analysis by sex, shift work status, and occupation

**Figure 2:**
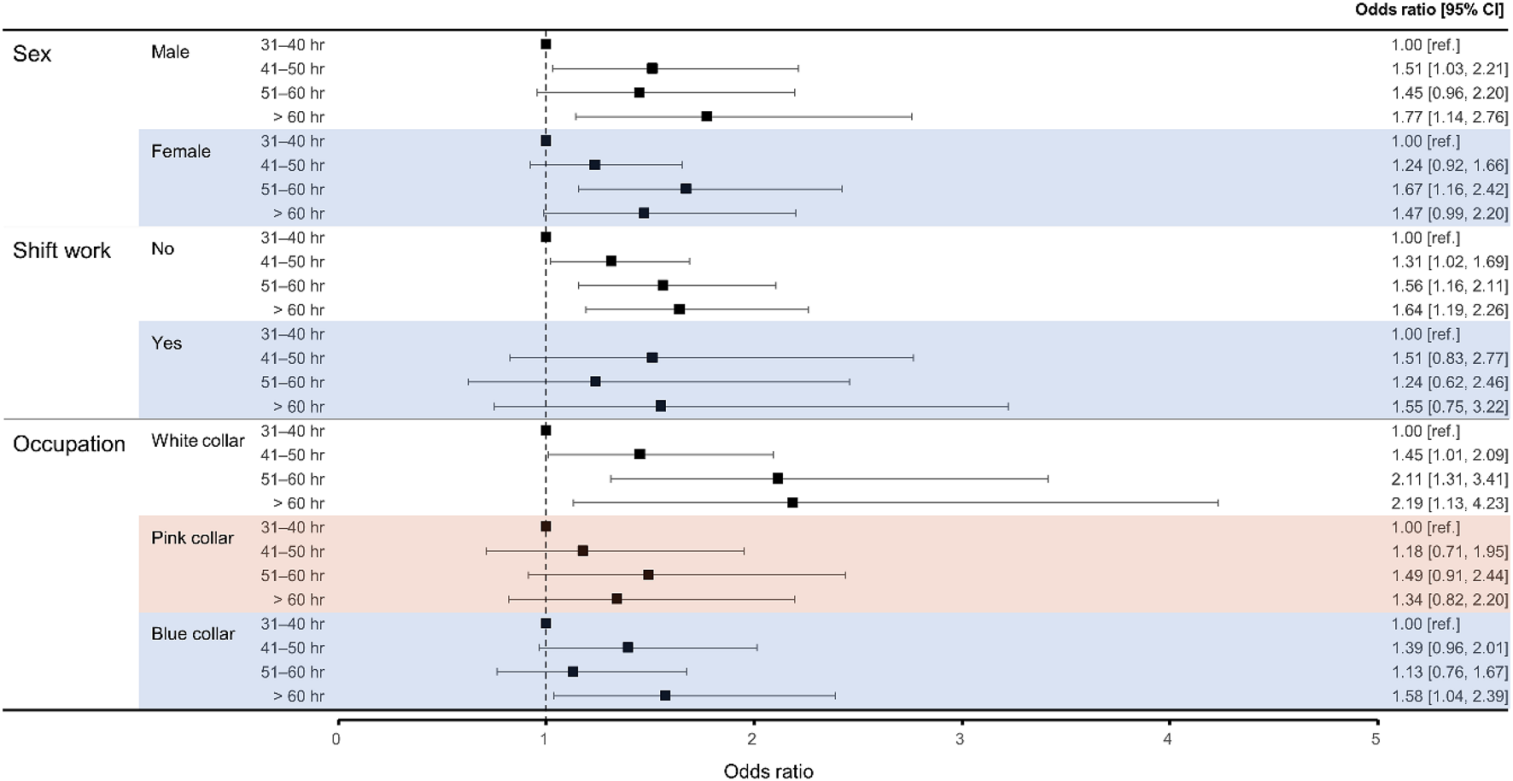
Associations of depressive mood with working hours according to subgroup. Model 3 was fully adjusted for age, sex, smoking status, alcohol consumption, educational level, household income, marital status, perceived health status, occupation, sleep duration, physical activity, and comorbidities. CI, confidence interval.

We explored the effects of sex, shift work, and occupation. In men, participants who worked > 60 h exhibited a higher risk of depressive mood, compared with participants who worked 31–40 h (OR 1.77, 95% CI 1.14–2.76). For non-shift work, longer working hours were strongly associated with depressive mood (ORs for 41–50, 51–60, and > 60 h: 1.13 [95% CI 1.02–1.69], 1.56 [95% CI 1.16–2.11], and 1.64 [95% CI 1.19–2.26], respectively). In terms of occupation, longer white-collar working hours were associated with higher risks of depressive mood (ORs for 41–50, 51–60, and > 60 h: 1.45 [95% CI 1.01–2.09], 2.11 [95% CI 1.31–3.41], and 2.19 [95% CI 1.13–4.23]). Blue-collar work > 60 h was strongly associated with depressive mood (OR 1.58, 95% CI 1.04–2.39) (Fig. 2).

**Figure 3:**
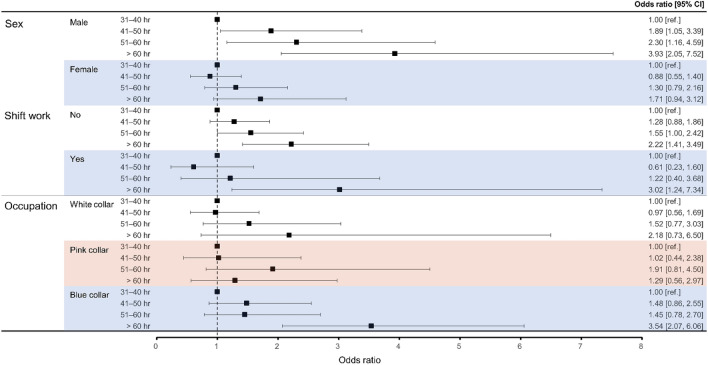
Associations of suicidal ideation with working hours according to subgroup. Model 3 was fully adjusted for age, sex, smoking status, alcohol consumption, educational level, household income, marital status, perceived health status, occupation, sleep duration, physical activity, and comorbidities. CI, confidence interval.

Suicidal ideation risk increased significantly with working hours in men (ORs for 41–50, 51–60, and > 60 h: 1.89 [95% CI 1.05–3.39], 2.30 [95% CI 1.16–4.59], and 3.93 [95% CI 2.05–7.52]). In the group working > 60 h, shift work featured a larger OR than did non-shift work (3.02 [95% CI 1.24–7.34] vs. 2.22 [95% CI 1.41–3.49]). Among occupations, blue-collar work > 60 h was associated with the highest risk of suicidal ideation (OR 3.54 [95% CI 2.07–6.06]) (Fig. 3).

## Discussion

We found that longer working hours were significantly associated with depressive mood and suicidal ideation in adults. The relationship with suicidal ideation was stronger in men who were blue-collar or shift workers.

Suicidal ideation is defined as any thoughts, images, beliefs, voices, or other cognitions reported by the individual about intentionally ending his or her own life^27^. Suicidal ideation can be presumed to exist when an individual has thought about suicide even if he or she has not seriously considered it, and various screening tools have been developed to measure the severity of suicidal ideation^28–30^. One of them, the Beck Depression Inventory-II (BDI-II), evaluates a patient's suicidal ideation on one of four scales; 0 ("I have no intention of committing suicide), 1 (I have thoughts of suicide, but I will not do it."), 2 ("I would like to commit suicide), and 3 (I would commit suicide if I had the chance"). In the KNHANES data we used, the question was asked about seriously considered suicidal ideation, so it could be considered to be equivalent to a level of 2 or higher on the BDI-II scale. A study of 6891 psychiatric outpatients by GK Brown et al. found that those with a BDI-II score of 2 or greater were 6.9 times more likely to commit suicide than those with less than score of 2^31^.

A recent study suggested new risk factors related to suicidal ideation through the IMV model of suicidal behavior^32^. The IMV model consists of three phases: a pre-motivational phase (background factors), a motivational phase (suicidal ideation/intention formation), and a volitional phase (behavioral enaction). The pre-motivational phase explains that background factors such as socioeconomic and environmental factors influence suicidal behavior^33^. In the motivational phase, it was argued that defeat and/or humiliation from which there is no perceived escape—a sense of entrapment is involved in the psychological processes for suicidal ideation to occur. In this process, it was explained that the presence or absence of threat to self-moderators (e.g. social problem-solving and coping) and motivational moderators (e.g. more positive future thoughts and goals) influence the development of suicidal ideations. In our study, we considered background factors (socioeconomic and environmental factors) as much as possible and tried to investigate the effect of long working hours, which could act as entrapment, on suicidal ideation.

Long working hours create distress and affect physical health because of their relationships with lack of exercise, more smoking, and more alcohol consumption^8,34–36^. Long hours also increase anxiety and depression^37^. Among United Kingdom public officials, individuals who worked more than 55 h were 1.66-fold more likely to have depression and 1.74-fold more likely to experience anxiety, compared with individuals who worked 35–40 h^38^. Similarly, in our study, the OR for depressive mood was 1.6-fold higher (95% CI 1.19–2.14) for participants who worked > 60 h, compared with participants who worked 31–40 h. Long working hours are also associated with suicidal risk; Kim et al. reported that suicidal ideation was 1.38-fold higher among individuals working > 60 h than among individuals working 40–51 h^39^. Yoon et al. reported an increased risk of suicidal ideation among individuals working ≥ 60 h compared with individuals working < 52 h^18^. In our study, compared with participants who worked 31–40 h, the OR for suicide was 2.3-fold greater for participants who worked > 60 h (95% CI 1.54–3.45).

Previous studies have shown that long working hours affected mental health more among women than among men^40,41^. We found that women who worked > 60 h exhibited a higher OR for depressive mood, compared with men. However, we found a relationship between suicidal ideation and working hours only in men. Shift work causes sleep problems because it does not synchronize with the 24-h circadian rhythm, thereby worsening mental health^42^. We found similar results. Among shift workers, participants who worked > 60 h exhibited a higher OR for suicidal ideation, compared with non-shift workers. In terms of occupation, Kim et al. reported that blue-collar workers exhibited higher depression scores than did white-collar workers^43^. In our study, the OR (3.54) for suicidal ideation was highest for blue-collar participants who worked > 60 h.

There are several possible explanations for blue-collar workers being more suicidal. Blue-collar workers are usually employed as one of the non-managerial occupations, and thus often have low autonomy and decision-making rights^44^. Blue-collar workers tend to be more stressed due to conflicts caused by problems such as vertical communication with managers^45^. Also, blue-collar workers show a more vulnerable health condition than white-collar workers, because they are much exposed to dangerous physical conditions such as hard physical work, noise, and chemicals, and tend to have relatively little interest in health^46–49^. Stress and physical illness are well known as major risk factors for suicidal behavior^33,50^.

In our study, the relationship between pink-collar occupation and suicidal ideation showed opposite results depending on whether environmental factors were adjusted or not. In model 1 (unadjusted) and model 2 (adjusted by age and sex), pink-collar occupation was positively related with suicidal ideation, whereas in model 3 (fully adjusted including environmental factors) it showed a negative relationship. This is because environmental factors such as smoking status, alcohol consumption, educational level, household income, marital status, perceived health status, sleep duration, physical activity, and comorbidities acted as mediators or confounders.

How do long working hours affect mental health? First, home rest time is shortened; workers do not fully recover from work-induced stress and fatigue^51^. Second, long working hours reduce sleep quality and volume; sleep problems are associated with depression and anxiety^52,53^. Furthermore, if a worker cannot control the workload (and thus may work overtime), an imbalance between effort and reward may develop. Stress increases if the work reward is perceived to be inadequate^54^. In this way, workers who work long hours may feel defeated and humiliated, with their self-moderators keep threatened, and eventually fall into entrapment^55^.

The principal strength of this study is the very large and nationally representative sample used for analysis. The results can be generalized to the entire adult population of Korea. To the best of our knowledge, this is the largest study thus far regarding the relationships of working hours with depressive mood and suicidal ideation in an Asian population. However, this work had several limitations. First, the KNHANES data were not collected for this study's purposes, so we were not able to go through the rigorous data collection strategies commonly implemented in established research studies. Second, because the study was cross-sectional in nature, we could not infer any causal relationship. Third, because all data were self-reported, recall bias may have influenced the analysis. For example, the number of working hours was measured via a single self-reported question. Though self-reported questionnaires had been proven useful in examining working hours at the population level in several studies, more objective measurements will be needed in further studies^8,18,54,56^. Fourth, as the questionnaire investigated the mental health of the past year, the specific point in time of the depressive symptoms or suicidal ideation was not clarified. Fifth, depressive mood and suicidal ideation were defined through yes/no dichotomous questions. Though various symptoms such as sadness, hopelessness, fear of the future, and the persistent thought that life is not worth living can indicate depressive mood, the questionnaire used in KNHANES data asked only the sadness or hopelessness^57^. Suicidal ideation may also vary in severity or definition depending on the measurement method^28–30^. Sixth, because ethnic and geographical characteristics may have affected the results, caution is needed when generalizing the results to non-Korean populations. A well-designed large-scale study is required.

## Conclusion

We studied a nationally representative Korean population. After adjustments for sociodemographic and mental health-related variables, we found that long working hours were strongly associated with depressive mood and suicidal ideation. Men, as well as shift and blue-collar workers, were more vulnerable to suicidal ideation. Working hours should be limited by law.

## Data Availability

The KCDC data are available at https://knhanes.cdc.go.kr/knhanes/sub03/sub03_02_05.do (accessed 8 April 2021). The raw data can be downloaded by anyone who meets the criteria specified by the KCDC.
